# Chest ultrasounds and X-rays compared in patients with acute dyspnea in an Emergency Department

**DOI:** 10.1186/2036-7902-6-S2-A1

**Published:** 2014-08-27

**Authors:** IF Martino, G Statti, F Fancoli, C Tinelli, MA Bressan

**Affiliations:** 1 Emergency Department, Scientific Direction, Policlinico San Matteo, Pavia, Italy

## Background

Dyspnea is one of the most frequent causes of access to the Emergency Department (ED). A major challenge for the emergency physician lies in differentiating diagnosis between cardiogenic and respiratory dyspnea. Currently, the instrument mainly used for the diagnosis of acute pulmonary disease is a chest X-ray: however, chest ultrasounds (US) are proving their potential in the diagnosis of acute dyspnea.

## Objective

*Primary objective*: to demonstrate diagnosis accuracy of chest USs using chest X-rays as a parameter for comparison in patients with acute dyspnea.

*Secondary objective:* to quantify savings in terms of time and resources.

## Patients and methods

We enrolled all patients over the age of 18 suffering from acute dyspnea, not consequent to trauma of the chest or pneumothorax. All patients were subjected to chest USs and chest X-rays (in double blind).

## Results

From January 1st to July 30th 2013, we enrolled 62 patients with acute dyspnea.

The concordance between the two methods was 97% (K value 0.9 = almost perfect). Regarding the diagnosis of pleural effusion, the concordance was 90% (K value 0.8=substantial agreement).

Regarding the savings:

- Time: the average time necessary to obtain an X-ray is 36 minutes; time for a chest US is the time required for the visit.

- Costs: the average cost of an X-ray is 37 Euros. Only in 2 cases was there no match, ideally giving a saving of € 2220 (60 X-rays X 37 Euros).

## Conclusion

Chest USs have a high reliability for diagnosis of acute dyspnea as compared to X-rays (more sensitive than X-rays for the diagnosis of pleural effusion). USs allow a saving in time and costs. Chest Uss should be considered an essential tool for the emergency physician.
